# Epidemiology and Economic Burden of Sleep Disorders in Europe

**DOI:** 10.1111/ene.70463

**Published:** 2026-02-14

**Authors:** Claudio L. A. Bassetti, Luisa S. Welter, Mateo Montes‐Martinez, Nikolai Mühlberger, Paul Boon, Thomas Berger, Günther Deuschl, Marjan Arvandi, Maria Konti, Maria Lolich, Evelina Pajediene, Rolf Fronczek, Elena Moro, Uwe Siebert, Richard Dodel

**Affiliations:** ^1^ Medical Faculty University of Bern and Neurology Department, Inselspital Bern Switzerland; ^2^ Department of Geriatric Medicine University Duisburg‐Essen Essen Germany; ^3^ Institute of Public Health, Medical Decision Making and Health Technology Assessment, Department of Public Health and Health Technology Assessment UMIT TIROL – University for Health Sciences and Technology Hall in Tirol Austria; ^4^ Department of Neurology 4Brain Ghent University Hospital & Ghent University Ghent Belgium; ^5^ Department of Neurology Medical University of Vienna Vienna Austria; ^6^ Department of Neurology Christian Albrecht University Kiel Germany; ^7^ European Academy of Neurology Vienna Austria; ^8^ Department of Neurology, Medical Academy Lithuanian University of Health Sciences Kaunas Lithuania; ^9^ Department of Neurology Leiden University Medical Centre Leiden the Netherlands; ^10^ Stichting Epilepsie Instellingen Nederlands (SEIN), Sleep‐Wake Centre Heemstede the Netherlands; ^11^ Division of Neurology Grenoble Institute of Neurosciences, CHU of Grenoble, Grenoble Alpes University Grenoble France; ^12^ Department of Epidemiology and Health Policy & Management Harvard T.H. Chan School of Public Health Boston Massachusetts USA; ^13^ Department of Health Policy & Management, Center for Health Decision Science Harvard T.H. Chan School of Public Health Boston Massachusetts USA; ^14^ Department of Radiology, Institute for Technology Assessment Massachusetts General Hospital, Harvard Medical School Boston Massachusetts USA

**Keywords:** cost‐of‐illness, epidemiology, insomnia, sleep apnea, sleep disorders

## Abstract

**Background:**

Sleep and sleep disorders (SD) have a major impact on brain (neurological and psychiatric), body and societal health. Despite this, the epidemiological and economic burden of SD have not been sufficiently analyzed. This study investigates the epidemiology and costs of SD across 47 European countries and identifies knowledge gaps in the literature.

**Methods:**

Systematic literature reviews on PubMed (between January 2010 and April 2023) and expert communications identified relevant epidemiological and cost‐of‐illness (COI) studies on five major SD: insomnia, obstructive sleep apnea (OSA), narcolepsy, restless legs syndrome (RLS), and REM sleep behavior disorder (RBD). Four epidemiological parameters, including prevalence, were investigated. Economic analyses stratified direct, indirect, and informal care costs, and employed an imputation procedure that accounts for several country‐specific economic factors. Costs were expressed as purchasing power parity (PPP)‐adjusted 2019 Euros.

**Results:**

Eleven COI and six epidemiological studies were identified. Estimated prevalence for OSA, insomnia, RLS, narcolepsy, and RBD in the adult population was 18%, 10%, 3%, 0.03%, and 0.009%, respectively. Economic data were exclusively available for high‐income Europe. OSA was the most costly SD (€184 billion), followed by insomnia (€158 billion), RLS (€79 billion), narcolepsy (€905 million), and RBD (€436 million). Direct and indirect costs contributed 48% and 52%, respectively, with no available data on informal care costs.

**Conclusions:**

The unexpected high prevalence and substantial economic burden associated with SD contrast with the universally neglected role of sleep health and SD in public health strategies. More research on the burden of SD is needed.

## Graphical abstract

We investigated the epidemiology and costs of five major sleep disorders in Europe using systematic literature reviews, data harmonization, and imputation. Estimated adult prevalence was 18% for OSA, 10% for insomnia, 3% for RLS, 0.03% for narcolepsy, and 0.009% for RBD. In 2019, OSA had the highest economic impact in high‐income Europe (€184 billion), followed by insomnia (€158 billion), RLS (€79 billion), RBD (€436 million), and narcolepsy (€905 million).

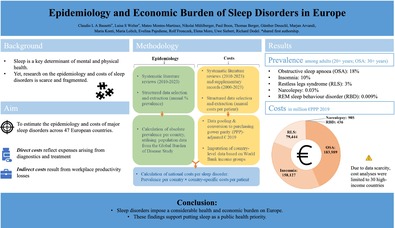

**Abbreviations**
COI: cost‐of‐illness
CRSD: circadian rhythm sleep disorders
EAN: European Academy of Neurology
GBD: global burden of disease
GDP: gross domestic product
GNI: gross national income
OSA: obstructive sleep apnea
PPP: purchasing power parity
RBD: rapid eye movement sleep behavior disorder
RLS: restless legs syndrome
SD: sleep disorders

## 1 Introduction

Sleep is an essential determinant of physical, brain (neurological and mental), and societal health [1, 2]. Sleep loss (insufficiency) and sleep disorders (SD) chronically affect 20%–30% of the population [3, 4] and have a tremendous impact on quality of life and productivity [5]. SD, also referred to as sleep–wake–circadian disorders, encompass a diverse range of clinical conditions that disrupt key physiological processes vital for memory, metabolism, and tissue repair, while also impairing daytime function, cognition, and mood. SD represent independent risk factors and/or modulators of several neurological (e.g., dementia, stroke, and Parkinsonism), psychiatric (e.g., depression), metabolic (e.g., diabetes), cardiovascular (e.g., hypertension), and neoplastic disorders [1, 2, 6, 7, 8].

A high economic burden of SD has been suggested by a few studies [5, 9, 10, 11]. However, SD are not incorporated into recent large‐scale systematic studies on the burden of brain diseases [12, 13]. This neglect may be due to the (incorrect) assumption that SD are usually secondary to an underlying other (somatic or psychiatric) condition, the fact that SD are managed by different specialists, and an overall underestimation (also by the medical community) of the frequency, health consequences, and societal relevance of SD [14].

The Cost of Illness in Neurology project (COIN‐Eu) was initiated in 2020 by the European Academy of Neurology (EAN) by one of the authors of this paper (C.L.A.B.) during his presidency with the aim of evaluating the economic burden of 12 groups of neurological disorders, including SD, in Europe.

As part of this initiative, the current study aims to (1) estimate the epidemiological and economic burden of SD across the 47 EAN member states in 2019, and (2) identify key research gaps in the epidemiological and cost‐of‐illness (COI) literature on SD.

## 2 Methods

### 2.1 Targeted Populations and Outcomes

This health‐economic research focuses on sleep disorders (SD) as defined by the International Classification of Sleep Disorders, Third Edition, Text Revision (ICSD‐3‐TR; AASM, 2023) [15]. Although insufficient sleep also affects nonclinical populations, this study adopts a disease‐oriented perspective, excluding nonclinical sleep issues like sleep loss. Initially, six SD categories were considered: insomnia, sleep‐related breathing disorders, sleep‐related movement disorders, central disorders of hypersomnolence, parasomnias, and circadian rhythm sleep disorders (CRSD). However, due to limited peer‐reviewed sources, the scope was narrowed to insomnia, obstructive sleep apnea (OSA), restless legs syndrome (RLS), narcolepsy (types 1 and 2), and rapid eye movement sleep behavior disorder (RBD). CRSD were excluded due to the absence of COI studies. Table 1 lists the included SD with their ICD‐10 codes.

**TABLE 1:** Five sleep disorders with ICD‐10 codes and annual prevalence.

Sleep disorder	ICD‐10 code	Average annual prevalence (%)
REM sleep behavior disorder (RBD)	G47.52	0.009 [16]
Narcolepsy	G47.41	0.03 [16, 17, 18]
Restless legs syndrome (RLS)	G25.81	3 [19]
Insomnia disorder	G47.00	10 [20]
Obstructive sleep apnea (OSA)	G47.33	18 [21]

*Note:* Assumed annual prevalence in adults (aged 20 years or older). For OSA, due to scarce data for the population 20+ years, we present the prevalence in persons aged 30 years or older. For narcolepsy and OSA, an average across countries is shown here, while country‐specific data were used in the cost calculations.

This study focuses on the adult population (20 years and older), as SD in minors differ in clinical presentation and treatment. The target region includes all 47 member countries of the European Academy of Neurology (EAN).

The epidemiological burden of SD was investigated by considering prevalence, incidence, mortality, and Disability‐Adjusted Life Years (DALY). The index year is 2019, chosen as the last year before the COVID‐19 pandemic, which may have disrupted typical patterns.

Three key economic components were analyzed: direct, indirect, and informal care costs, accounting for multiple payer perspectives (e.g., healthcare systems, patients, employers, caregivers, and taxpayers) to comprehensively assess the economic impact of SD. Direct costs include medical expenses for diagnosis and treatment, and nonmedical direct costs like social services. Indirect costs cover productivity losses due to absenteeism or presenteeism, while informal care refers to unpaid assistance with Activities of Daily Living (ADL). A detailed breakdown of these three major cost categories is documented in Table S1. Intangible costs (e.g., emotional well‐being), and lost tax revenue were excluded.

### 2.2 Epidemiological Data

Epidemiological data were retrieved through a systematic PubMed search, targeting review articles published from January 2010 to March 2023, and expert recommendations. The search algorithm combined title and MeSH terms (see Table S2A). A backward citation approach was employed to identify relevant primary studies. Inclusion and exclusion criteria are detailed in Table S3A.

Data were extracted into a structured matrix, documenting methodological features of the respective studies, country, and SD subgroup. Epidemiological measures were extracted as relative figures, such as percentages or rates. Extraction was conducted by one researcher and verified by a second, with discrepancies resolved by a third (L.S.W., M.M.‐M., V.D.). To compensate for data scarcity in the targeted European region, global epidemiological sources were included. The extraction matrix was divided into separate tables for each of the six SD. Then, data were scrutinized for suitability, considering factors such as study quality and case identification methods. This evaluation led to a refinement of the study's score. For example, the focus was narrowed from general insomnia to insomnia disorder to ensure consistent case identification (see Section 3). The final selection also considered the availability of matching economic data. To illustrate, for parasomnias, while prevalence data existed for various subtypes, only one COI was available, focusing on the RBD subtype. Therefore, prevalence data on RBD were selected.

For countries with multiple data sources, relative measures were averaged, and simple averages across countries substituted missing country‐level data. Relative epidemiological outcomes (percentages, rates) were converted into absolute, country‐specific figures for 2019 using population data from the 2021 Global Burden of Disease (GBD) Study [13]. These calculations covered the adult population (20+ years; OSA: 30+ years) and the working‐age population (20–64 years; OSA: 30–64 years).

### 2.3 Economic Data

Economic data were sourced through three methods. First, a systematic PubMed review identified economic articles published between January 2010 and April 2023, using a search algorithm refined for sensitivity and specificity (see Table S2B). Eligible publications were identified through title screening and full‐text review, and relevant reviews were examined for eligible primary studies. To supplement the limited data available since 2010, articles from a pilot study covering 2000–2010 were included. Lastly, additional records were considered based on expert recommendations. Inclusion criteria are outlined in Table S3B.

Cost data were extracted following a per‐patient, annual format. The extraction template, refined during a pilot phase, included key fields such as region, publication details, cost year, currency, and detailed cost component breakdowns. To ensure consistency and best use of the available literature, an all‐cause approach was adopted, focusing on costs for individuals with SD rather than disease‐attributable (excess) costs. Two researchers independently extracted selected sources, with a third resolving any discrepancies.

A second refinement step after the initial extraction led to the exclusion of less methodologically suitable studies. Empirical data were prioritized over modeling, and studies with small sample sizes (*n* < 14) or limited cost assessments (e.g., only drug costs) were excluded. Some sampling biases (such as specific age groups or comorbidities) were tolerated if no other study was available.

The extracted cost data were standardized to 2019 purchasing power parity (PPP) EUR using World Bank data. Nonnational currencies were reconverted, inflated using the Consumer Price Index (CPI), and converted to EUR 2019 PPP. Country‐specific costs were pooled per category using the median. For direct nonmedical costs, medians were derived and combined with direct medical costs to estimate total direct costs.

Missing data were imputed using methods adapted from Gustavsson et al. [9] and Begley et al. [22]. Countries were grouped by income level (lower‐middle, upper‐middle, high) based on 2019 World Bank GNI categories. Medians for each cost type within income groups were weighted with country‐specific factors, such as health expenditures relative to GDP and GDP per capita, to reflect economic context. National direct costs were calculated by multiplying per‐patient costs by the country‐specific prevalence for the adult population (20+ years; OSA: 30+ years), while indirect costs used prevalence within the working‐age population (20–64 years; OSA: 30–64 years). Per capita costs were calculated by dividing total costs by population size (GBD population data). Per‐patient costs across SD were calculated using a weighted average based on the prevalence of each individual SD. These synthesis and imputation procedures will be detailed in a separate report.

## 3 Results

### 3.1 Characterization of Included Sources

Out of 982 identified records, six sources were selected for epidemiological analysis (see Figure S1 for the PRISMA flowchart). Although the search targeted prevalence, incidence, mortality, and DALYs, no eligible studies on mortality or DALYs were found. Six sources provided prevalence data (narcolepsy: *n* = 3; insomnia, OSA, RLS, and RBD: *n* = 1, respectively). Two incidence studies on narcolepsy were selected, and no viable source was identified for CRSD. The initial data extraction revealed considerable variability due to methodological differences, with insomnia prevalence ranging from 5% with syndrome‐based case identifications [23] to 40% with symptom‐oriented operationalizations [24].

For the final analysis, high‐quality sources using precise case definitions were selected. For insomnia, we relied on a review by Morin and Jarrin [20], who used stringent classification criteria to estimate a 10% prevalence rate of insomnia disorder. For RLS, we selected Broström et al. [19], a recently published, comprehensive systematic review, meta‐analysis, and meta‐regression, that applied several bias corrections. For OSA, Benjafield et al. [21] provided country‐specific prevalence data aligned with the American Academy of Sleep Medicine's scoring criteria. Data for an apnea‐hypopnea index (AHI) of ≥ 15 were selected to match our economic data. As no suitable reviews for narcolepsy or RBD were found, primary studies were utilized. Details on all selected epidemiological sources are provided in Table S4A.

From 552 initially identified economic sources, our final estimations were based on 11 studies from high‐income countries (Figure S2 provides the PRISMA flowchart). The selection process distilled economic studies on narcolepsy (*n* = 4), OSA (*n* = 3), insomnia (*n* = 2), RBD, and RLS (*n* = 1, each). No eligible source was identified for CRSD. Data were reported for direct medical costs (10 data points), indirect costs (8), and direct nonmedical costs (1), with no data on informal care costs. Nine studies used observational designs—based on national registries (*n* = 4), clinical recruitment (*n* = 4), and a postal survey (*n* = 1). Two economic sources constituted cost‐effectiveness analyses. Table S4B outlines key methodological features and extracted cost figures from all included economic sources.

### 3.2 Prevalence of Sleep Disorders

Table 1 shows the prevalence by SD, with OSA being the most common (18%) and RBD being the rarest (0.0087%). Prevalence rate estimates are largely homogenous across countries due to reliance on imputation. Country‐specific prevalence data are documented in the Supporting Information for insomnia (Table S6A), RBD (Table S6B), OSA (Table S6C), RLS (Table S6D), and narcolepsy (Table S6E).

### 3.3 Costs of Sleep Disorders

Here, we present the costs of SD in high‐income Europe. Due to substantial gaps in the COI literature, no reliable estimates were available for middle‐income countries. Comprehensive cost tables, including national estimates and country‐specific per‐patient estimates for each SD, are provided in Table S6A–E.

**FIGURE 1:**
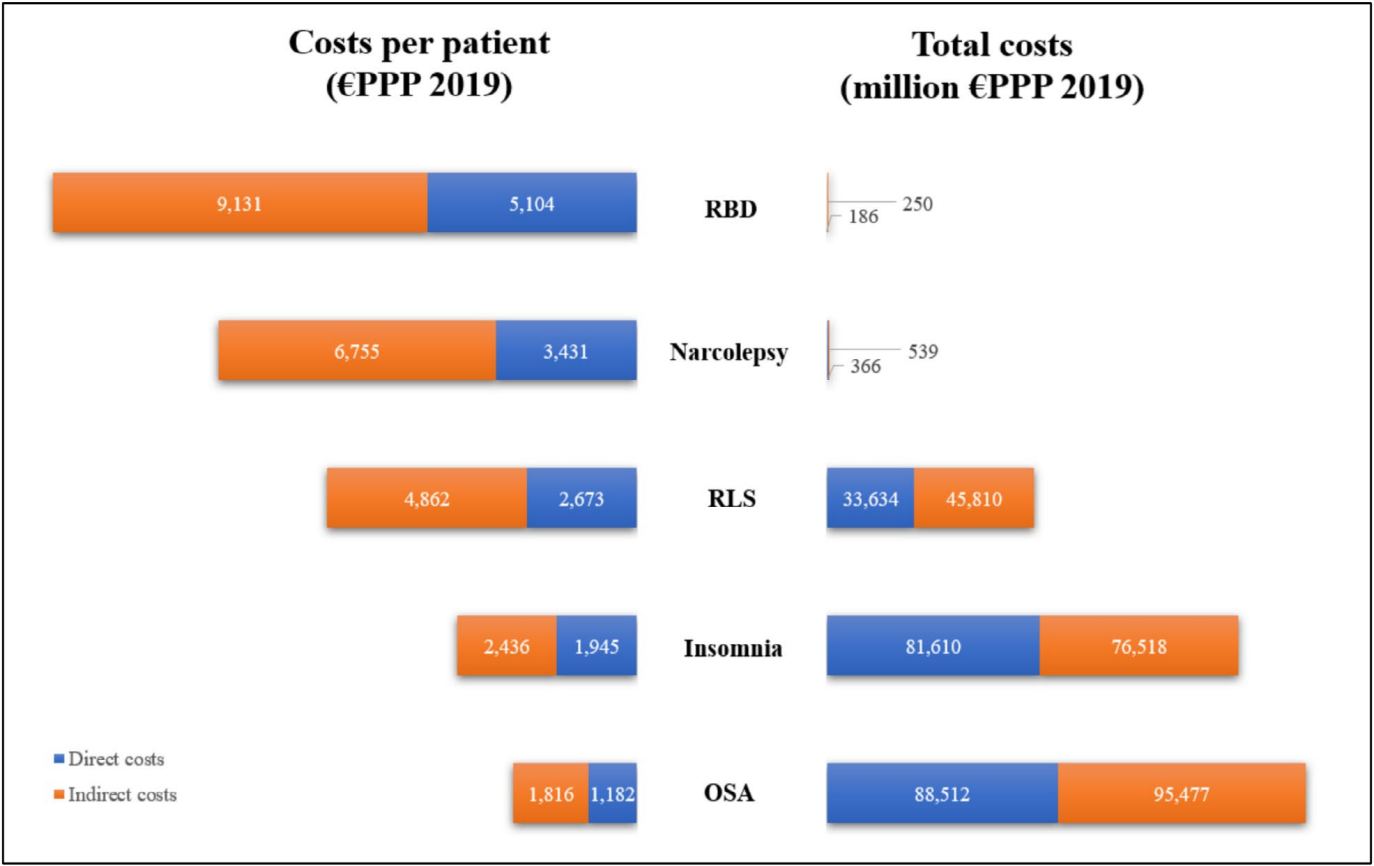
The costs of sleep disorders. This figure juxtaposes the costs per patient with the total costs for five sleep disorders: REM sleep behavior disorder (RBD), narcolepsy, restless legs syndrome (RLS), insomnia, and obstructive sleep apnea (OSA). Costs per patient represent the average per‐patient costs per SD, weighted by country prevalence. Total costs reflect the collective national costs across 30 European high‐income countries. PPP, purchasing power parity. Median costs per patient: RBD indirect: 9023, direct: 4599; Narcolepsy indirect: 5868, direct: 2476; RLS indirect: 4827, direct: 2382; Insomnia indirect: 2438, direct: 1753; OSA indirect: 1734, direct: 1023.

**FIGURE 2:**
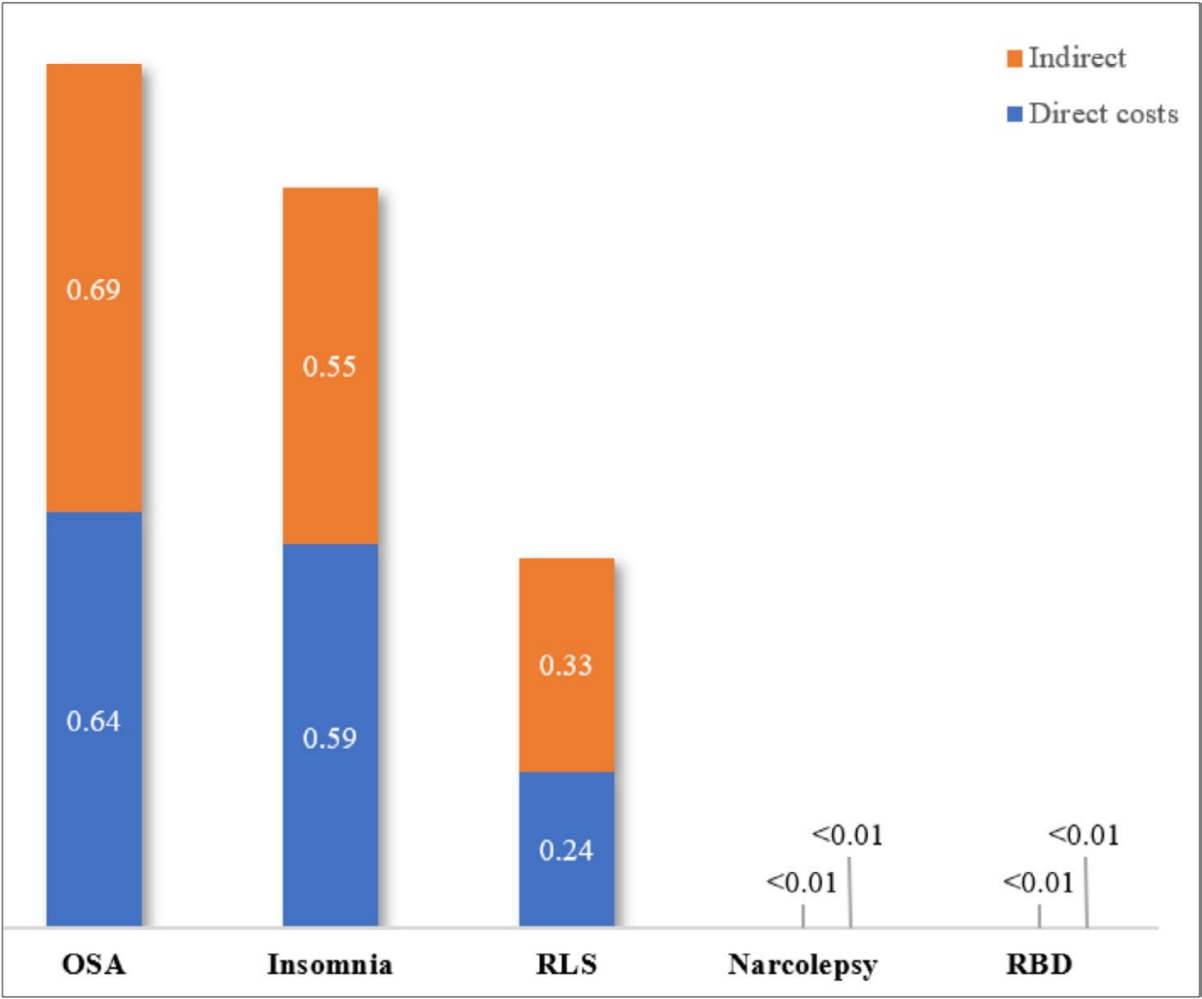
Costs per sleep disorder relative to collective GDP in high‐income Europe (%). Column chart illustrating the direct and indirect costs of obstructive sleep apnea (OSA), insomnia, restless legs syndrome (RLS), narcolepsy, and REM sleep behavior disorder (RBD), as a percentage of the collective Gross Domestic Product (GDP) of 30 European high‐income countries.

In 2019, OSA had the highest societal costs (€184 billion), followed by insomnia (€158 billion), RLS (€79 billion), narcolepsy (€905 million), and RBD (€436 million). These cost differences largely reflect prevalence rates. Per‐patient costs ranged from €3002 for OSA to €14,234 for RBD (Figure 1). OSA and insomnia costs equalled 1.32% and 1.14% of the region's combined GDP (Figure 2).

Direct and indirect costs account for 48% and 52% of the total SD burden, respectively, though the distribution varies by disorder. For narcolepsy, 40% of costs stem from direct expenses (e.g., diagnosis and treatment), while 60% arise from productivity losses due to workplace absences and reduced labor force participation.

The aggregated costs of SD in high‐income Europe are estimated at €422.9 billion, equivalent to 3% of the region's collective GDP. This figure needs to be interpreted cautiously due to the potential for double‐counting. Germany bore the highest societal costs (€106 billion), followed by France (€68 billion), the UK (€39 billion), and Italy (€36 billion), reflecting their large populations. Per‐capita costs ranged from €372 in Croatia to €1855 in Switzerland, with a median of €620 (Table S5).

## 4 Discussion

This study is the first to systematically assess the epidemiological and economic burden for a spectrum of SD in Europe since 2010. Providing comprehensive estimates, it facilitates comparisons between disorders, cost types, and European countries, and provides a much‐needed update to previous estimates and studies focused on single SD.

### 4.1 Epidemiology of Sleep Disorders

SD affect a large portion of the population. However, studies on the epidemiology of SD suffer from several major limitations. First, only a few studies assessed the prevalence of any SD in a given population. One study from Denmark encompassing the entire population and using nationwide data from healthcare and socioeconomic registries reported a 7% prevalence of major SD^22^. A Dutch study of a nationally representative sample of 2089 individuals using a set of 48 questions reported an overall prevalence of SD of 32% [3]. Second, the case identification methods used in epidemiological sleep research vary among studies. As a consequence, prevalence rates for chronic insomnia range from 4.7% (ICD‐10) to 22.1% (DSM‐5), and prevalence rates for narcolepsy range from 1.05 to 79.40 per 100,000 persons [25, 26]. Studies on OSA vary in AHI cutoffs and criteria for hypopnea [21, 27]. Additionally, prevalence studies often focus on middle‐aged and elderly individuals, while a prevalence as high as 16% has also been suggested for young adults [28]. Third, reports from Canada and Germany indicate a post‐COVID increase in SD prevalence [20, 29].

In addition to limited data on the prevalence of SD, other key epidemiological measures have received even less attention. First, disability‐adjusted life years (DALYs), quantifying the morbidity‐ and mortality‐related societal burden, are profoundly under‐assessed for SD. For numerous other brain diseases, DALYs are regularly published [13]. Few studies have explored the disability burden from environmentally induced sleep disturbances in Europe [30, 31, 32]. Second, country‐specific impacts of SD on mortality are inadequately studied despite their association with adverse health outcomes. Recent population‐based research links numerous SD to increased mortality [33]. Third, limited research on SD incidence further hinders the understanding of regional trends and locally relevant causes.

### 4.2 Cost of Sleep Disorders

The estimated overall cost of SD in Europe amounted to nearly €423 billion in 2019. This best estimate, while still subject to uncertainties arising from data scarcity, equates to a 3% taxation on the collective GDP in high‐income Europe. It accounts for approximately 25% of the region's costs from neurological diseases [34]. The highest costs were associated with OSA (1.32% of GDP), while RBD had the highest per‐patient cost (€14,234). The per‐capita costs vary regionally (from €372 in Croatia to €1855 in Switzerland), reflecting differences in their economic structures, including price levels and average salaries. Direct and indirect costs contributed roughly equally, with some variation by disorder.

Despite this considerable financial burden, profound gaps exist in the economic literature on SD. Contemporary large‐scale studies on the costs of brain disorders often do not include SD [12], and original research is fragmented. Middle‐income countries are particularly underrepresented, despite their large populations (42% of the European population investigated here). Further, the extent of research varies between cost types. Although absenteeism costs are somewhat studied, presenteeism costs (i.e., costs from reduced alertness and daytime sleepiness during work hours) remain overlooked. Informal care costs for SD are unknown in Europe, though studies from Australia suggest they contribute less than 1% to the overall costs of OSA and insomnia [11, 35]. However, disorders like narcolepsy may require assistance in daily activities, the financial implications of which are underexplored.

The last estimation of SD‐related costs, spanning multiple European countries and considering various cost elements, dates back to Gustavsson et al. [9]. Total costs were estimated at €35.4 billion in 2010 for four SD (hypersomnia, nonorganic insomnia, narcolepsy, and sleep apnea) in 30 countries (EU‐27 plus Iceland, Norway, and Switzerland). This figure equates to nearly €40 billion in 2019 (€PPP, adjusted using the Euro area harmonized index of consumer prices) [36]. Despite a moderate overall agreement, notable differences include a large discrepancy in sleep apnea prevalence (3% in Gustavsson et al. vs. 18% in ours) and per‐patient costs for insomnia (€153 in 2010 vs. €4381 in 2019). Key methodological differences contribute to these discrepancies, including unequal disease classifications, our broader selection of primary studies, and our focus on all‐cause rather than excess costs. Comparisons between the 2010 and 2019 estimates should thus be made cautiously, and deviations between past and current estimates underscore the need for additional investigation.

A more recent registry‐based study from Denmark documents the costs across five SD in 2015 [37]. All‐cause direct costs are reported at €6045 per patient, while excess direct costs were reported at €2659, similar to our estimate for Denmark (€2555 in 2019). Indirect per‐person costs were much higher than our estimate (€8691 in 2015 vs. €3075 in 2019).

Other recent reports focus on individual SD, cost types, or countries. RAND Europe investigated the financial burden of insomnia in 16 Western countries [10] and the cost of insufficient sleep across five OECD countries [38]. Their findings on insomnia prevalence align with ours, reporting 8% for chronic insomnia and 14% for clinical insomnia. Another investigation across five European high‐income countries reports a slightly lower prevalence for chronic insomnia disorder (6%) [39]. Both studies confirm the high prevalence of insomnia symptoms at 34% and 21%, respectively, showcasing the spectrum of symptomology. RAND Europe documents annual GDP losses from lost workplace productivity due to chronic insomnia between 0.64% and 1.31%, an estimate higher than our 0.49%–0.89% range. This discrepancy likely originates from a lack of scientific data on presenteeism costs in Europe. The report also reveals that adults would trade 14% of their household income to alleviate insomnia symptoms, suggesting a large subjective burden.

### 4.3 Limitations of This Study

Several limitations must be noted for an accurate interpretation of our figures. Primarily, current estimates are compromised by scarce and unstandardized primary research. While incorporating a wide array of sources enhances comprehensive estimations, it also introduces a degree of blurriness due to differing criteria for case identification and cost assessments. Current research gaps also concern the comorbidity between different SD. In epidemiological research, it remains unclear what proportion of the population is affected by any sleep disorder, as opposed to a particular SD. Thus, the prevalence of SD is likely lower than the sum of individual SD prevalence. A similar challenge arises in many cost‐of‐illness studies, where per‐patient costs may be influenced by comorbid (sleep) disorders and may thus not represent costs specifically attributable to a single SD. When summing up costs across different SD, these uncertainties introduce a risk of double counting. Consequently, aggregate estimates of the total costs across SD should be interpreted with caution. Further, a bias toward treated patient populations raises concerns about generalizability from primary studies, as treated patients often exhibit more severe symptoms, skewing per‐person estimates upward. When scaled to the national level, these figures represent the costs of treating all (more severely affected) patients, rather than actual healthcare expenditures. Many individuals with SD remain untreated due to factors like lack of awareness, stigma, or barriers to accessing care. Consequently, SD are often underdiagnosed, misdiagnosed, and undertreated [40, 41]. More population‐based data are needed to estimate the real‐world costs of SD. Lastly, our imputation procedure is constrained by its reliance on country groupings based on GNI, resulting in missing estimates for 36% of the countries originally considered, particularly in middle‐income regions. While alternative methods like nearest‐neighbor approaches bypass the dependence on categorization, they risk comparing dissimilar societies.

### 4.4 Implications of This Study

The implications of this study extend to clinicians, researchers, policymakers, and the general society. More awareness about sleep as a determinant of health, the overall burden of SD, and the increasing possibilities offered by modern sleep medicine to promote the prevention, early diagnosis, and treatment of SD is needed. This will alleviate the very high healthcare costs and work productivity losses presented in this paper and previous [5, 41].

Considering the high prevalence of sleep–wake complaints and SD, new approaches, including the collaboration with and education of nonsleep specialists and the use of novel screening approaches, are needed [2, 14]. Furthermore, public health programs should in the future include the promotion of sleep health and the prevention of sleep disorders [42]. Finally, more research is needed to understand the role of sleep in the complex interaction between internal and external exposome, which eventually determines individual health [43].

## 5 Conclusions

The current study demonstrates a substantial epidemiological and economic burden related to sleep disorders (SD). Taking into account the increasing evidence for an important role of sleep on health and well‐being, the data presented here call for the recognition of sleep health and SD as a major public health priority.

More research is however needed to reduce the uncertainty associated with current best estimates. Informed health policies would greatly benefit from incorporating SD into multinational burden estimates such as the GBD Study [13].

## Author Contributions

Ideas: C.L.A.B., R.D. Conceptualization: C.L.A.B., R.D., U.S., G.D., P.B., T.B., N.M., L.S.W., M.M.‐M. Funding acquisition: R.D., U.S. Development of methodology: R.D., U.S., C.L.A.B., N.M., L.S.W., M.M.‐M. Investigation: L.S.W., M.M.‐M., N.M., C.L.A.B. Literature search – design: R.D., L.S.W., M.M.‐M. Literature search – execution: L.S.W., M.M.‐M., C.L.A.B. Data curation: L.S.W., M.M.‐M., N.M., C.L.A.B. Data analysis: N.M., M.A., M.M.‐M., L.S.W., U.S., R.D. Data validation: C.L.A.B., E.P., R.F., L.S.W., M.M.‐M., N.M., M.A., G.D., P.B., T.B., R.D., U.S. Writing – original draft preparation: L.S.W., C.L.A.B. Writing – review and editing: all authors. Visualization/figures: L.S.W., M.M.‐M. Supervision: R.D., C.L.A.B. Project administration: M.K., M.L.

## Funding

The European Academy of Neurology commissioned this study (coin‐health.eu). The funding organization had no role in the study design, data collection, data analysis, data interpretation, report writing, or decision to publish.

## Ethics Statement

This research utilized publicly available data without collecting individual patient data for the analysis. Consequently, it did not meet the regulatory definition of human participant research and was exempt from review by an institutional review board or ethics committee.

## Conflicts of Interest

Several authors currently hold or have held leading and representative positions in neurological committees and institutions (C.L.A.B., P.B., E.M., T.B., G.D., E.P., R.F.).

## Supporting information


**Data S1:** ene70463‐sup‐0001‐Supinfo.docx.

## Data Availability

The data that support the findings of this study are available in the Supporting Information of this article.
