# Supplementary material for: Epidemiology and Economic Burden of Sleep Disorders in Europe
Source: Eur J Neurol. 2026 Feb 14;33(2):e70463. doi: 10.1111/ene.70463 (PMC12905625; doi:10.1111/ene.70463)
Supplement: Supplementary file 1 — Data S1: ene70463‐sup‐0001‐Supinfo.docx. [file ENE-33-e70463-s001.docx]

**Supplementary Material**

[Table 1: Breakdown of Cost Elements 2](#_Toc203032036)

[Table 2: PubMed-based Search Algorithms 2](#_Toc203032037)

[Table 3: Study Inclusion and Exclusion Criteria 3](#_Toc203032038)

[Table 4: Selected Sources 7](#_Toc203032039)

[Table 5: Annual Per-Capita Costs of Sleep Disorders By Country 13](#_Toc203032040)

[Table 6: Costs and Prevalence of Five Major Sleep Disorders Per Country 14](#_Toc203032041)

[Figure 1: PRISMA Flow Chart: Record Selection Process (Epidemiology) 4](#_Toc203032042)

[Figure 2: PRISMA Flow Chart: Record Selection Process (Cost of Illness) 5](#_Toc203032043)

Table 1: Breakdown of Cost Elements

| **Direct costs** | Medical: inpatient care, outpatient care, medications, medical procedures and devices  Non-medical: social services, adaptations, transportation, additional costs, such as long-term care |
| --- | --- |
| **Indirect costs** | Costs related to productivity losses: short-term absence from work (sick leave, absenteeism), presenteeism, foregone income, early retirement, premature mortality |
| **Informal care costs** | Assistance in Activities of Daily Living (ADL) and Instrumental Activities of Daily Living (iADL) |

Table detailing cost subcomponents considered within the three main cost categories.

Table 2: PubMed-based Search Algorithms

A) Epidemiology Query

| **Disease-related search terms** | ("Sleep Wake Disorders"[MeSH Terms] OR "sleep disorder*"[Title] OR "insomn*"[Title] OR "sleep related breathing"[Title] OR "sleep apnea"[Title] OR "hypersomn*"[Title] OR "Circadian Rhythm sleep"[Title] OR "parasomn*"[Title] OR "restless legs syndrome"[Title] OR "Nocturnal Myoclonus Syndrome"[Title] OR "Sleep Bruxism"[Title] OR "sleep talking"[Title] OR "Kleine-Levin Syndrome"[Title] OR "somnambulism"[Title]) |
| --- | --- |
| **Epidemiological search terms** | AND ("Epidemiology"[MeSH Terms] OR "epidemiolog*"[Title] OR "Incidence"[MeSH Terms] OR "Incidence"[Title] OR "Prevalence"[MeSH Terms] OR "Prevalence"[Title] OR "Mortality"[MeSH Terms] OR "Mortality"[Title] OR "deaths"[Title] OR "Disability-Adjusted Life Years"[MeSH Terms] OR "Disability-Adjusted Life Years"[Title] OR "daly*"[Title] OR "Years of Life Lost"[Title] OR "yll"[Title] OR "Years Lived with Disability"[Title] OR "yld"[Title]) |
| **Publication type and time** | AND (review[Publication Type] OR Meta-Analysis[Publication Type] OR Systematic Review[Publication Type] OR review[Title] OR meta-analys*[Title])  AND (2010:2023[pdat]) |

B) Cost-of-Illness Query

| **Disease-related search terms** | ("Sleep Wake Disorders"[MeSH Terms] OR "sleep disorder*"[Title] OR "insomn*"[Title] OR "sleep related breathing"[Title] OR "sleep apnea"[Title] OR "osas"[Title] OR "osa"[Title]  OR “catathrenia”[Title] OR "hypersomn*"[Title] OR “narcolepsy”[Title] OR "Circadian Rhythm sleep"[Title] OR "parasomn*"[Title] OR "restless legs syndrome"[Title] OR “RLS”[Title] OR "Nocturnal Myoclonus Syndrome"[Title] OR "Sleep Bruxism"[Title] OR "sleep talking"[Title] OR "somnambulism"[Title]) |
| --- | --- |
| **Economic search terms** | AND ("cost of illness"[MeSH Terms] OR "health expenditures"[MeSH Terms] OR "Health care costs"[MeSH Terms] OR "cost*"[Title] OR "economic*"[Title] OR "financial*"[Title] OR "expenditure*"[Title] OR "expense*"[Title] OR "monetary"[Title]) |
| **Publication time** | AND (2010:2023[pdat]) |

Tables segmenting the search algorithms used to identify epidemiological (Table 2A) and economic (Table 2B) data into disease-related, outcome-related, and publication-related components.

Table 3: Study Inclusion and Exclusion Criteria

A) Epidemiological Sources

| Inclusion criteria | Exclusion criteria |
| --- | --- |
| Study population: adult patients (20 years or older); patients receiving standard treatment | only reporting data for minors (=< 19 years old); samples receiving atypical treatments |
| Article language: English, German, Spanish | Published in other languages |
| Only peer-reviewed research and review articles were considered | Grey literature including policy literature, working papers, and government documents. |

B) Economic Sources

| Inclusion criteria | Exclusion criteria |
| --- | --- |
| Sources reporting diagnosis-related and post-diagnosis costs | Sources reporting only pre-diagnosis costs |
| Cost categories: direct, indirect, and informal care costs | Sources reporting only intangible costs, costs associated with lost tax revenue, costs to caregivers, or costs due to workplace or vehicle-related accidents |
| Scope and granularity of cost assessment: studies reporting data for at least one of the main cost categories (direct, indirect, informal care costs) | Narrow assessments, such as investigations merely focusing on drug costs were ignored if in the presence of a more comprehensive alternative; estimates of the total costs without or with unclear breakdown into direct, indirect and/or informal care costs were also excluded. |
| Peer-reviewed research articles | Grey literature including policy literature, working papers, and government documents. |
| Full-text available in the English language | Full-text not available in English |
| Observational study designs; randomized controlled trials (RCT); cost-effectiveness analysis (CEA); cost-utility analysis (CUA) | Case reports, modelling studies |
| Sample size: n>=14 | n<14 |

Tables listing the inclusion and exclusion criteria guiding the selection of epidemiological (Table 3A) and economic (Table 3B) studies during systematic literature review.

Figure 1: PRISMA Flow Chart: Record Selection Process (Epidemiology)


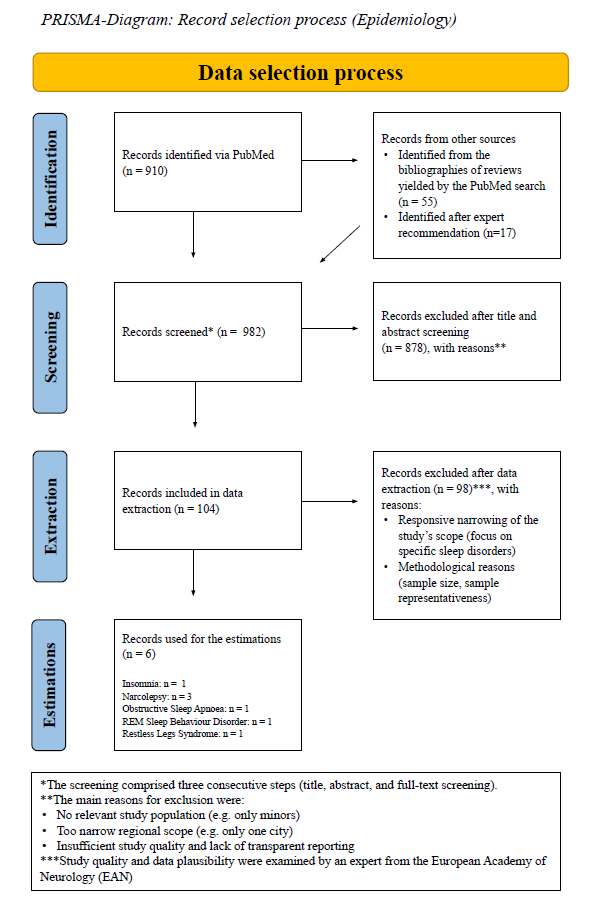


Flow diagram outlining the selection process for epidemiological studies, adhering to Preferred Reporting Items for Systematic reviews and Meta-Analyses (PRISMA) guidelines.

Figure 2: PRISMA Flow Chart: Record Selection Process (Cost of Illness)


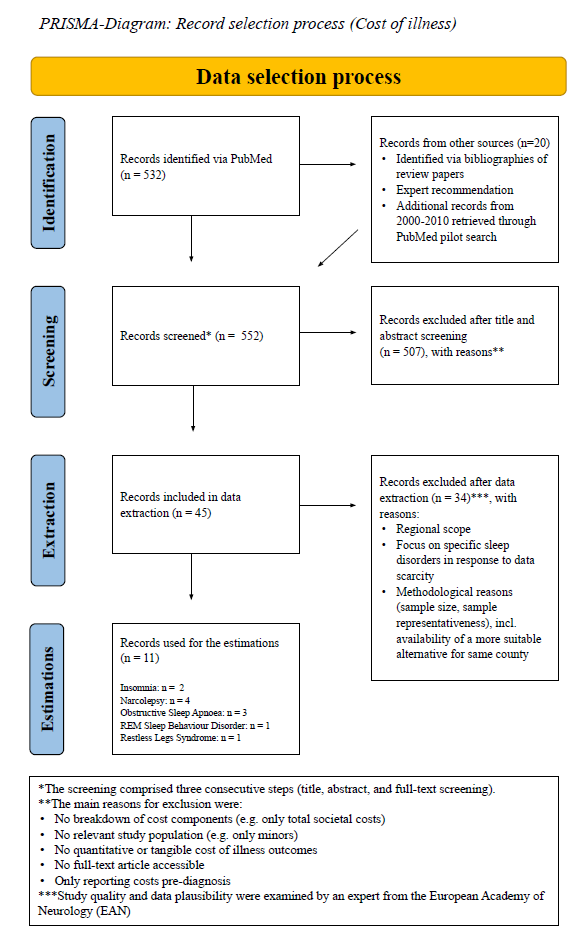


Flow diagram outlining the selection process for economic studies, adhering to Preferred Reporting Items for Systematic reviews and Meta-Analyses (PRISMA) guidelines.

Table 4: Selected Sources

A) Selected Epidemiological Sources

| Sleep disorder | Authors | Country | Measure | Extracted figure | Methodological key considerations  Article type (AT); Sources (S); Calculation method (C); Disregards (D); Weighting (W); Sample characteristics (SC); Others (O) |
| --- | --- | --- | --- | --- | --- |
| Narcolepsy | Feketeova et al., 2020^1^ | Slovakia | Prevalence | 0.001047 % | AT: Primary study.  S: Data were sourced through records from specialized centres.  C: We converted the values to percentages by dividing by 10.000. |
| Narcolepsy | Feketeova et al., 2020^1^ | Slovakia | Incidence | 0.000057 % | AT: Primary study.  S: Data were sourced through records from specialized centres.  C: We converted the values to percentages by dividing by 10.000. |
| Narcolepsy | Silber et al., 2002†^2^ | United States of America | Prevalence | 0.0563 % | AT: Primary study.  S: Data were sourced through a records-linkage system of the Rochester Epidemiology  Project n in Olmsted County. Minnesota.  C: We converted the values to percentages by dividing by 1.000. |
| Narcolepsy | Silber et al., 2002†^2^ | United States of America | Incidence | 0.00137 % | AT: Primary study.  S: Data were sourced through a records-linkage system of the Rochester Epidemiology  Project n in Olmsted County. Minnesota.  C: We converted the values to percentages by dividing by 1.000. |
| Narcolepsy | Acquavella et al., 2020^3^ | United States of America | Prevalence | 0.0443 % | AT: Primary study.  S: Data were retrieved from nationwide (USA) medical/prescription claims (Symphony Health).  C: We converted the values to percentages by dividing by 1.000.  W: Different types of insurance were considered.  O: Data were extracted for the most recent year with reported data (2016). |
| Obstructive sleep apnoea | Benjafield et al., 2019^4^ | 165 countries | Prevalence | 18.1249 %‡ | AT: Review article with a conversion algorithm to adapt data to AAMS 2012 criteria and an imputation procedure. assigning data to countries with similar characteristics.  SC: Focus on the population aged 30-69 years.  O: We chose to exclusively consider the population with an AHI of >=15. |
| Restless legs syndrome | Broström et al., 2023^5^ | global | Prevalence | 3.0000 % | AT: Systematic review. meta-analysis and meta-regression.  SC: Focus on the adult population.  O: We extracted the corrected prevalence. |
| Insomnia | Morin & Jarrin, 2022^6^ |  | Prevalence | 10.000 % | AT: Review paper.  SC: Focus on the adult population.  O: We extracted the global value for insomnia disorder. |
| Rapid eye movement (REM) sleep behaviour disorder | Acquavella et al., 2020^3^ | United States of America | Prevalence | 0.0087 % | AT: Primary study.  S: Data were retrieved from nationwide (USA) medical/prescription claims (Symphony Health).  C: We converted the values to percentages by dividing by 1.000.  W: Different types of insurance were considered.  O: Data were extracted for the most recent year with reported data (2016). |
| Circadian rhythm sleep disorders | N/A | N/A | N/A | N/A | N/A |

Summary of key methodological characteristics, extraction decisions, and data obtained from the epidemiological sources included in the final calculations. Full citations are provided in the reference list below Table 4B.

† identified via review article: Wang. Yiping et al. “Heterogeneity in Estimates of Incidence and Prevalence of Narcolepsy: A Systematic Review and Meta-Regression Analysis.” Neuroepidemiology vol. 56.5 (2022): 319-332. doi:10.1159/000525282. PMID: 35820399 ^7^

‡ This value represents the naive average across countries with available estimates from the review. Country-specific estimates were extracted and used in subsequent calculation.

B) Selected Economic Sources

| Sleep disorder | Authors | Country | DMC | DNMC | IC | Currency | Index year | Sample size | Key considerations: Sources (S); Calculation method (C); Disregards (D); Weighting (W); Sample characteristics (SC); Others (O) |
| --- | --- | --- | --- | --- | --- | --- | --- | --- | --- |
| Insomnia | Godet-Cayré et al., 2006^8^ | France |  |  | 2,511 | EUR | 2004 | 369 | SC: The people recruited are physicians who worked at least a 28-hour week (80% of full-time employment in France) C: Costs due to absenteeism combining four different payer perspectives. |
| Insomnia | Dragioti et al., 2018^9^ | Sweden | 4,580 |  |  | EUR | 2012 | 845 | S: The participants completed a postal survey assessing the required data for the study. SC: The sample consisted of people over 64 years old with chronic pain. W: The costs were weighted considering the subthreshold, moderate clinical, and severe clinical insomnia subgroups. |
| Obstructive sleep apnoea | Jennum & Kjellberg, 2011^10^ | Denmark | 2,984 |  | 2,273 | EUR | 2006 | 19,438 | S: Data was retrieved from the Danish National Patient Registry (1998-2006). This is a nationally representative study. D: Publicly funded transfer payments were disregarded. |
| Obstructive sleep apnoea | Pelletier-Fleury et al., 2004^11^ | France | 839 |  |  | EUR | 2003 | 171 | S: The results are part of a cost-effectiveness analysis and randomized controlled trial. SC: Patients with moderate-to-severe obstructive sleep apnoea syndrome. C: The costs were weighted considering the apnoea/hypopnoea index under 30 and equal or over 30 subsamples and their immediate or delayed treatment subgroups. |
| Obstructive sleep apnoea | McMillan et al., 2015^12^ | United Kingdom | 1,376 |  |  | GBP | 2012 | 278 | S: The results are part of a cost-effectiveness analysis and randomized controlled trial. SC: Only patients aged 65 or older with newly diagnosed obstructive sleep apnoea syndrome were included. W: The costs were weighted considering the patients treated with continuous positive airway pressure receiving best supportive care or only best supportive care. |
| Restless legs syndrome | Dodel et al., 2010^13^ | Germany | 3,117 |  | 5,233 | EUR | 2006 | 519 | S: Patients were recruited from several centres in Germany and filled out a questionnaire. C: The costs were calculated for 3 months. They were multiplied by 4 to annualize them. |
| Narcolepsy | Jennum et al., 2009^14^ | Denmark | 4,163 |  | 7,490 | EUR | 2005 | 459 | S: Data was retrieved from the Danish National Patient Registry (1998-2005). This is a nationally representative study. C: The indirect costs encompass foregone earnings, pension and sick pay. |
| Narcolepsy | Jennum et al., 2012^15^ | Denmark | 4,236 |  | 9,214 | EUR | 2009 | 816 | S: Data was retrieved from the Danish National Patient Registry (1997-2009). This is a nationally representative study. C: The indirect costs encompass foregone earnings, pension and sick pay. |
| Narcolepsy | Dodel et al., 2004^16^ | Germany | 1,560 |  | 11,620 | EUR | 2002 | 75 | S: Patients were recruited at the Hephata Klinik between 1999 and 2002 and were contacted by phone. |
| Narcolepsy | Ingravallo et al., 2012^17^ | Italy | 8,646 |  | 984 | EUR | 2011 | 100 | S: Patients were recruited at the outpatient Clinic for Narcolepsy of the Sleep Disorders Centre of the University of Bologna. SC: Narcolepsy with cataplexy. |
| REM sleep behaviour disorder | Frandsen, Rune et al., 2021^18^ | Denmark | 7,291 | 455 | 12,590 | EUR | 2009 | 246 | S: Data was retrieved from the Danish National Patient Registry (2006-2016). This is a nationally representative study. |

Summary of key methodological characteristics, extraction decisions, and cost data obtained from the primary economic sources included in the final cost calculations. Full citations are provided in the reference list below.

**References**

1. Feketeova E, Tormasiova M, Klobučníková K, et al. Narcolepsy in Slovakia - Epidemiology, clinical and polysomnographic features, comorbid diagnoses: a case-control study. *Sleep Med*. 2020;67:15-22. doi:10.1016/j.sleep.2019.10.012

2. Silber MH, Krahn LE, Olson EJ, Pankratz VS. The epidemiology of narcolepsy in Olmsted County, Minnesota: a population-based study. *Sleep*. 2002;25(2):197-202. doi:10.1093/sleep/25.2.197

3. Acquavella J, Mehra R, Bron M, Suomi JMH, Hess GP. Prevalence of narcolepsy and other sleep disorders and frequency of diagnostic tests from 2013-2016 in insured patients actively seeking care. *J Clin Sleep Med JCSM Off Publ Am Acad Sleep Med*. 2020;16(8):1255-1263. doi:10.5664/jcsm.8482

4. Benjafield AV, Ayas NT, Eastwood PR, et al. Estimation of the global prevalence and burden of obstructive sleep apnoea: a literature-based analysis. *Lancet Respir Med*. 2019;7(8):687-698. doi:10.1016/S2213-2600(19)30198-5

5. Broström A, Alimoradi Z, Lind J, Ulander M, Lundin F, Pakpour A. Worldwide estimation of restless legs syndrome: a systematic review and meta-analysis of prevalence in the general adult population. *J Sleep Res*. 2023;32(3):e13783. doi:10.1111/jsr.13783

6. Morin CM, Jarrin DC. Epidemiology of Insomnia: Prevalence, Course, Risk Factors, and Public Health Burden. *Sleep Med Clin*. 2022;17(2):173-191. doi:10.1016/j.jsmc.2022.03.003

7. Wang Y, Chen Y, Tong Y, Li C, Li J, Wang X. Heterogeneity in Estimates of Incidence and Prevalence of Narcolepsy: A Systematic Review and Meta-Regression Analysis. *Neuroepidemiology*. 2022;56(5):319-332. doi:10.1159/000525282

8. Godet-Cayré V, Pelletier-Fleury N, Le Vaillant M, Dinet J, Massuel MA, Léger D. Insomnia and absenteeism at work. Who pays the cost? *Sleep*. 2006;29(2):179-184. doi:10.1093/sleep/29.2.179

9. Dragioti E, Bernfort L, Larsson B, Gerdle B, Levin LÅ. Association of insomnia severity with well-being, quality of life and health care costs: A cross-sectional study in older adults with chronic pain (PainS65+). *Eur J Pain Lond Engl*. 2018;22(2):414-425. doi:10.1002/ejp.1130

10. Jennum P, Kjellberg J. Health, social and economical consequences of sleep-disordered breathing: a controlled national study. *Thorax*. 2011;66(7):560-566. doi:10.1136/thx.2010.143958

11. Pelletier-Fleury N, Meslier N, Gagnadoux F, et al. Economic arguments for the immediate management of moderate-to-severe obstructive sleep apnoea syndrome. *Eur Respir J*. 2004;23(1):53-60. doi:10.1183/09031936.03.00066903

12. McMillan A, Bratton DJ, Faria R, et al. A multicentre randomised controlled trial and economic evaluation of continuous positive airway pressure for the treatment of obstructive sleep apnoea syndrome in older people: PREDICT. *Health Technol Assess Winch Engl*. 2015;19(40):1-188. doi:10.3310/hta19400

13. Dodel R, Happe S, Peglau I, et al. Health economic burden of patients with restless legs syndrome in a German ambulatory setting. *PharmacoEconomics*. 2010;28(5):381-393. doi:10.2165/11531030-000000000-00000

14. Jennum P, Knudsen S, Kjellberg J. The economic consequences of narcolepsy. *J Clin Sleep Med JCSM Off Publ Am Acad Sleep Med*. 2009;5(3):240-245.

15. Jennum P, Ibsen R, Petersen ER, Knudsen S, Kjellberg J. Health, social, and economic consequences of narcolepsy: a controlled national study evaluating the societal effect on patients and their partners. *Sleep Med*. 2012;13(8):1086-1093. doi:10.1016/j.sleep.2012.06.006

16. Dodel R, Peter H, Walbert T, et al. The socioeconomic impact of narcolepsy. *Sleep*. 2004;27(6):1123-1128. doi:10.1093/sleep/27.6.1123

17. Ingravallo F, Gnucci V, Pizza F, et al. The burden of narcolepsy with cataplexy: how disease history and clinical features influence socio-economic outcomes. *Sleep Med*. 2012;13(10):1293-1300. doi:10.1016/j.sleep.2012.08.002

18. Frandsen R, Asah C, Ibsen R, Kjellberg J, Jennum PJ. Health, social, and economic consequences of rapid eye movement sleep behavior disorder: a controlled national study evaluating societal effects. *Sleep*. 2021;44(2):zsaa162. doi:10.1093/sleep/zsaa162

Table 5: Annual Per-Capita Costs of Sleep Disorders By Country

| Country | Population in 2019 | Cost per capita (€ PPP 2019) |
| --- | --- | --- |
| Austria | 8.900.697 | 1.136 |
| Belgium | 11.414.814 | 817 |
| Croatia | 4.252.705 | 372 |
| Cyprus | 1.319.377 | 567 |
| Czechia | 10.646.244 | 527 |
| Denmark | 5.800.319 | 1.347 |
| Estonia | 1.314.646 | 446 |
| Finland | 5.530.695 | 1.016 |
| France | 66.143.082 | 1.024 |
| Germany | 84.955.598 | 1.246 |
| Greece | 10.316.652 | 448 |
| Hungary | 9.688.911 | 448 |
| Iceland | 344.347 | 854 |
| Ireland | 4.903.561 | 739 |
| Israel | 9.283.686 | 442 |
| Italy | 35.693.965 | 598 |
| Latvia | 1.130.621 | 394 |
| Lithuania | 1.685.211 | 452 |
| Luxembourg | 396.087 | 1.353 |
| Netherlands | 10.066.030 | 1.107 |
| Norway | 5.346.385 | 1.019 |
| Poland | 23.894.666 | 412 |
| Portugal | 6.334.578 | 539 |
| Romania | 11.467.773 | 431 |
| Slovakia | 3.435.912 | 568 |
| Slovenia | 1.252.784 | 784 |
| Spain | 27.985.046 | 642 |
| Sweden | 5.775.301 | 816 |
| Switzerland | 8.776.629 | 1.855 |
| United Kingdom | 67.250.112 | 576 |

Table presenting population size and per-capita costs of five sleep disorders across 30 high-income European countries in 2019. Population data are sourced from the 2021 Global Burden of Disease Study. PPP: purchasing power parity. Table 6: Costs and Prevalence of Five Major Sleep Disorders Per Country

A) Insomnia

| EAN member country | Annual direct (a) costs per patient (€ PPP 2019) | Annual indirect (c) costs per patient (€ PPP 2019) | Annual total costs per patient  (€ PPP 2019) | Number of prevalent cases 2019 (adult population, 20+ years) | % working age (20-64 years) | Annual direct (a) costs per country (million € PPP 2019) | Annual indirect (c) costs per country (million € PPP 2019) | Annual total costs per country (million € PPP 2019) | GDP 2019 (million € PPP 2019) | Annual total costs related to GDP (% of GDP 2019) |
| --- | --- | --- | --- | --- | --- | --- | --- | --- | --- | --- |
| Albania |  |  |  | 205,500 | 81 |  |  |  | 10,710 |  |
| Armenia |  |  |  | 225,727 | 83 |  |  |  | 9,471 |  |
| Austria(a,c) | 2,131 | 3,195 | 5,326 | 716,196 | 77 | 1,526 | 1,753 | 3,279 | 309,190 | 1.06 |
| Azerbaijan |  |  |  | 731,495 | 91 |  |  |  | 33,500 |  |
| Belarus |  |  |  | 741,256 | 80 |  |  |  | 44,791 |  |
| Belgium(a,c) | 2,176 | 2,976 | 5,152 | 886,539 | 75 | 1,929 | 1,990 | 3,919 | 372,618 | 1.05 |
| Bosnia and Herzegovina |  |  |  | 270,080 | 78 |  |  |  | 14,049 |  |
| Bulgaria |  |  |  | 563,066 | 74 |  |  |  | 47,924 |  |
| Croatia(a,c) | 1,425 | 978 | 2,403 | 342,837 | 75 | 489 | 250 | 739 | 43,343 | 1.71 |
| Cyprus(a,c) | 1,431 | 1,877 | 3,308 | 104,089 | 82 | 149 | 161 | 310 | 18,042 | 1.72 |
| Czechia(a,c) | 1,600 | 1,510 | 3,110 | 846,362 | 75 | 1,354 | 954 | 2,308 | 175,623 | 1.31 |
| Denmark(a,c) | 2,033 | 3,802 | 5,835 | 449,962 | 75 | 915 | 1,277 | 2,192 | 240,956 | 0.91 |
| Estonia(a,c) | 1,374 | 1,495 | 2,869 | 103,677 | 75 | 142 | 116 | 258 | 21,614 | 1.20 |
| Finland(a,c) | 1,869 | 3,103 | 4,972 | 436,447 | 72 | 816 | 969 | 1,785 | 186,726 | 0.96 |
| France(a) | 2,258 | 2,854 | 5,112 | 5,022,410 | 74 | 11,341 | 10,545 | 21,886 | 1,897,662 | 1.15 |
| Georgia |  |  |  | 274,072 | 79 |  |  |  | 12,149 |  |
| Germany(a,c) | 2,389 | 2,986 | 5,374 | 6,912,615 | 74 | 16,512 | 15,225 | 31,738 | 2,703,880 | 1.17 |
| Greece(a,c) | 1,601 | 1,221 | 2,822 | 836,563 | 73 | 1,339 | 742 | 2,081 | 142,736 | 1.46 |
| Hungary(a,c) | 1,297 | 1,071 | 2,368 | 779,950 | 75 | 1,011 | 627 | 1,639 | 114,038 | 1.44 |
| Iceland(a,c) | 1,764 | 4,393 | 6,157 | 25,566 | 80 | 45 | 90 | 135 | 17,264 | 0.78 |
| Ireland(a,c) | 1,364 | 5,164 | 6,528 | 356,951 | 80 | 487 | 1,479 | 1,966 | 277,689 | 0.71 |
| Israel(a,c) | 1,524 | 2,836 | 4,360 | 599,759 | 81 | 914 | 1,383 | 2,297 | 279,879 | 0.82 |
| Italy(a,c) | 1,770 | 2,149 | 3,919 | 4,948,672 | 72 | 8,760 | 7,669 | 16,429 | 1,398,664 | 1.18 |
| Kazakhstan |  |  |  | 1,209,169 | 89 |  |  |  | 126,332 |  |
| Kyrgyzstan |  |  |  | 392,836 | 92 |  |  |  | 6,169 |  |
| Latvia(a,c) | 1,343 | 1,145 | 2,488 | 152,147 | 74 | 204 | 129 | 334 | 23,883 | 1.40 |
| Lithuania(a,c) | 1,431 | 1,250 | 2,681 | 224,032 | 75 | 321 | 211 | 531 | 38,074 | 1.40 |
| Luxembourg(a,c) | 1,097 | 7,186 | 8,283 | 48,601 | 81 | 53 | 285 | 338 | 48,557 | 0.70 |
| Montenegro |  |  |  | 46,941 | 81 |  |  |  | 3,854 |  |
| Netherlands(a,c) | 2,069 | 3,348 | 5,417 | 1,338,831 | 75 | 2,770 | 3,370 | 6,140 | 632,951 | 0.97 |
| North Macedonia |  |  |  | 172,567 | 83 |  |  |  | 8,766 |  |
| Norway(a,c) | 2,149 | 4,831 | 6,980 | 409,409 | 77 | 880 | 1,531 | 2,411 | 281,597 | 0.86 |
| Poland(a,c) | 1,317 | 1,002 | 2,318 | 3,071,609 | 78 | 4,044 | 2,394 | 6,437 | 414,498 | 1.55 |
| Portugal(a,c) | 1,946 | 1,489 | 3,435 | 868,461 | 73 | 1,690 | 943 | 2,633 | 166,887 | 1.58 |
| Republic of Moldova |  |  |  | 293,466 | 82 |  |  |  | 8,325 |  |
| Romania(a,c) | 1,173 | 827 | 2,000 | 1,515,670 | 76 | 1,778 | 948 | 2,726 | 174,559 | 1.56 |
| Russian Federation |  |  |  | 11,227,052 | 81 |  |  |  | 1,177,395 |  |
| Serbia |  |  |  | 708,423 | 77 |  |  |  | 35,823 |  |
| Slovakia(a,c) | 1,421 | 1,237 | 2,658 | 432,331 | 79 | 614 | 425 | 1,039 | 73,518 | 1.41 |
| Slovenia(a,c) | 1,741 | 1,660 | 3,401 | 167,052 | 75 | 291 | 208 | 499 | 37,782 | 1.32 |
| Spain(a,c) | 1,865 | 1,887 | 3,752 | 3,698,763 | 76 | 6,898 | 5,282 | 12,180 | 969,613 | 1.26 |
| Sweden(c) | 2,551 | 3,314 | 5,865 | 785,784 | 73 | 2,005 | 1,914 | 3,919 | 371,261 | 1.06 |
| Switzerland(a,c) | 2,306 | 5,367 | 7,673 | 703,943 | 77 | 1,623 | 2,898 | 4,521 | 501,642 | 0.90 |
| Türkiye |  |  |  | 5,746,863 | 87 |  |  |  | 528,462 |  |
| Ukraine |  |  |  | 3,522,136 | 79 |  |  |  | 107,011 |  |
| United Kingdom(a,c) | 2,074 | 2,727 | 4,801 | 5,164,238 | 76 | 10,709 | 10,748 | 21,457 | 1,986,804 | 1.08 |
| Uzbekistan |  |  |  | 2,099,096 | 92 |  |  |  | 41,660 |  |
| **Total** |  |  |  |  |  | **81,610** | **76,518** | **158,127** | **13,921,550** | **1.14** |

B) Rapid Eye Movement (REM) Sleep Behaviour Disorder

| EAN member country | Annual direct (a) costs per patient (€ PPP 2019) | Annual indirect (c) costs per patient (€ PPP 2019) | Annual total costs per patient  (€ PPP 2019) | Number of prevalent cases 2019 (adult population, 20+ years) | % working age (20-64 years) | Annual direct (a) costs per country (million € PPP 2019) | Annual indirect (c) costs per country (million € PPP 2019) | Annual total costs per country (million € PPP 2019) | GDP 2019 (million € PPP 2019) | Annual total costs related to GDP (% of GDP 2019) |
| --- | --- | --- | --- | --- | --- | --- | --- | --- | --- | --- |
| Albania |  |  |  | 179 | 80 |  |  |  | 10,710 |  |
| Armenia |  |  |  | 196 | 84 |  |  |  | 9,471 |  |
| Austria(a,c) | 5,592 | 12,183 | 17,775 | 623 | 77 | 3 | 6 | 9 | 309,190 | 0.00 |
| Azerbaijan |  |  |  | 636 | 91 |  |  |  | 33,500 |  |
| Belarus |  |  |  | 645 | 80 |  |  |  | 44,791 |  |
| Belgium(a,c) | 5,711 | 11,348 | 17,059 | 771 | 75 | 4 | 7 | 11 | 372,618 | 0.00 |
| Bosnia and Herzegovina |  |  |  | 235 | 78 |  |  |  | 14,049 |  |
| Bulgaria |  |  |  | 490 | 73 |  |  |  | 47,924 |  |
| Croatia(a,c) | 3,740 | 3,731 | 7,471 | 298 | 75 | 1 | 1 | 2 | 43,343 | 0.00 |
| Cyprus(a,c) | 3,757 | 7,158 | 10,914 | 91 | 82 | 0 | 1 | 1 | 18,042 | 0.01 |
| Czechia(a,c) | 4,199 | 5,758 | 9,957 | 736 | 75 | 3 | 3 | 6 | 175,623 | 0.00 |
| Denmark | 6,695 | 10,882 | 17,578 | 391 | 75 | 3 | 3 | 6 | 240,956 | 0.00 |
| Estonia(a,c) | 3,607 | 5,700 | 9,307 | 90 | 74 | 0 | 0 | 1 | 21,614 | 0.00 |
| Finland(a,c) | 4,906 | 11,833 | 16,738 | 380 | 72 | 2 | 3 | 5 | 186,726 | 0.00 |
| France(a,c) | 5,926 | 9,853 | 15,780 | 4,369 | 74 | 26 | 32 | 58 | 1,897,662 | 0.00 |
| Georgia |  |  |  | 238 | 79 |  |  |  | 12,149 |  |
| Germany(a,c) | 6,269 | 11,386 | 17,655 | 6,014 | 74 | 38 | 51 | 88 | 2,703,880 | 0.00 |
| Greece(a,c) | 4,201 | 4,658 | 8,859 | 728 | 73 | 3 | 2 | 6 | 142,736 | 0.00 |
| Hungary(a,c) | 3,403 | 4,084 | 7,487 | 679 | 75 | 2 | 2 | 4 | 114,038 | 0.00 |
| Iceland(a,c) | 4,630 | 16,753 | 21,383 | 22 | 82 | 0 | 0 | 0 | 17,264 | 0.00 |
| Ireland(a,c) | 3,580 | 19,691 | 23,271 | 311 | 80 | 1 | 5 | 6 | 277,689 | 0.00 |
| Israel(a,c) | 3,999 | 10,816 | 14,815 | 522 | 81 | 2 | 5 | 7 | 279,879 | 0.00 |
| Italy(a,c) | 4,646 | 8,193 | 12,839 | 4,305 | 72 | 20 | 25 | 45 | 1,398,664 | 0.00 |
| Kazakhstan |  |  |  | 1,052 | 89 |  |  |  | 126,332 |  |
| Kyrgyzstan |  |  |  | 342 | 92 |  |  |  | 6,169 |  |
| Latvia(a,c) | 3,526 | 4,366 | 7,892 | 132 | 74 | 0 | 0 | 1 | 23,883 | 0.00 |
| Lithuania(a,c) | 3,755 | 4,768 | 8,523 | 195 | 75 | 1 | 1 | 1 | 38,074 | 0.00 |
| Luxembourg(a,c) | 2,878 | 27,403 | 30,282 | 42 | 81 | 0 | 1 | 1 | 48,557 | 0.00 |
| Montenegro |  |  |  | 41 | 80 |  |  |  | 3,854 |  |
| Netherlands(a,c) | 5,429 | 12,769 | 18,198 | 1,165 | 75 | 6 | 11 | 18 | 632,951 | 0.00 |
| North Macedonia |  |  |  | 150 | 83 |  |  |  | 8,766 |  |
| Norway(a,c) | 5,639 | 18,424 | 24,063 | 356 | 78 | 2 | 5 | 7 | 281,597 | 0.00 |
| Poland(a,c) | 3,455 | 3,820 | 7,275 | 2,672 | 78 | 9 | 8 | 17 | 414,498 | 0.00 |
| Portugal(a,c) | 5,108 | 5,677 | 10,785 | 756 | 73 | 4 | 3 | 7 | 166,887 | 0.00 |
| Republic of Moldova |  |  |  | 255 | 82 |  |  |  | 8,325 |  |
| Romania(a,c) | 3,079 | 3,153 | 6,232 | 1,319 | 76 | 4 | 3 | 7 | 174,559 | 0.00 |
| Russian Federation |  |  |  | 9,768 | 81 |  |  |  | 1,177,395 |  |
| Serbia |  |  |  | 616 | 77 |  |  |  | 35,823 |  |
| Slovakia(a,c) | 3,730 | 4,716 | 8,446 | 376 | 80 | 1 | 1 | 3 | 73,518 | 0.00 |
| Slovenia(a,c) | 4,568 | 6,330 | 10,899 | 145 | 75 | 1 | 1 | 1 | 37,782 | 0.00 |
| Spain(a,c) | 4,894 | 7,198 | 12,092 | 3,218 | 76 | 16 | 18 | 33 | 969,613 | 0.00 |
| Sweden(a,c) | 5,825 | 12,638 | 18,462 | 684 | 73 | 4 | 6 | 10 | 371,261 | 0.00 |
| Switzerland(a,c) | 6,052 | 20,469 | 26,520 | 612 | 77 | 4 | 10 | 13 | 501,642 | 0.00 |
| Türkiye |  |  |  | 5,000 | 87 |  |  |  | 528,462 |  |
| Ukraine |  |  |  | 3,064 | 79 |  |  |  | 107,011 |  |
| United Kingdom(a,c) | 5,442 | 10,401 | 15,843 | 4,493 | 76 | 24 | 36 | 60 | 1,986,804 | 0.00 |
| Uzbekistan |  |  |  | 1,826 | 92 |  |  |  | 41,660 |  |
| **Total** |  |  |  |  |  | **186** | **250** | **436** | **13,921,550** | **0.00** |

C) Obstructive Sleep Apnoea

| EAN member country | Annual direct (a) costs per patient (€ PPP 2019) | Annual indirect (c) costs per patient (€ PPP 2019) | Annual total costs per patient  (€ PPP 2019) | Number of prevalent cases 2019 (adult population, 20+ years) | % working age (20-64 years) | Annual direct (a) costs per country (million € PPP 2019) | Annual indirect (c) costs per country (million € PPP 2019) | Annual total costs per country (million € PPP 2019) | GDP 2019 (million € PPP 2019) | Annual total costs related to GDP (% of GDP 2019) |
| --- | --- | --- | --- | --- | --- | --- | --- | --- | --- | --- |
| Albania |  |  |  | 558,004 | 76 |  |  |  | 10,710 |  |
| Armenia |  |  |  | 457,304 | 79 |  |  |  | 9,471 |  |
| Austria(a,c) | 1,304 | 2,340 | 3,644 | 1,707,923 | 72 | 2,228 | 2,884 | 5,111 | 309,190 | 1.65 |
| Azerbaijan |  |  |  | 1,334,498 | 89 |  |  |  | 33,500 |  |
| Belarus |  |  |  | 866,260 | 77 |  |  |  | 44,791 |  |
| Belgium(a,c) | 1,332 | 2,180 | 3,512 | 1,171,661 | 71 | 1,561 | 1,809 | 3,369 | 372,618 | 0.90 |
| Bosnia and Herzegovina |  |  |  | 654,493 | 74 |  |  |  | 14,049 |  |
| Bulgaria |  |  |  | 1,390,684 | 70 |  |  |  | 47,924 |  |
| Croatia(a,c) | 872 | 717 | 1,589 | 351,524 | 70 | 307 | 177 | 484 | 43,343 | 1.12 |
| Cyprus(a,c) | 876 | 1,375 | 2,251 | 142,005 | 78 | 124 | 153 | 277 | 18,042 | 1.54 |
| Czechia(a,c) | 979 | 1,106 | 2,085 | 1,220,889 | 71 | 1,196 | 956 | 2,152 | 175,623 | 1.23 |
| Denmark | 2,744 | 2,090 | 4,834 | 1,060,386 | 69 | 2,910 | 1,537 | 4,447 | 240,956 | 1.85 |
| Estonia(a,c) | 841 | 1,095 | 1,936 | 122,247 | 70 | 103 | 94 | 197 | 21,614 | 0.91 |
| Finland(a,c) | 1,144 | 2,273 | 3,417 | 1,090,635 | 66 | 1,248 | 1,647 | 2,894 | 186,726 | 1.55 |
| France(c) | 974 | 1,893 | 2,867 | 15,469,551 | 69 | 15,070 | 20,161 | 35,231 | 1,897,662 | 1.86 |
| Georgia |  |  |  | 238,967 | 75 |  |  |  | 12,149 |  |
| Germany(a,c) | 1,462 | 2,187 | 3,649 | 19,403,361 | 69 | 28,371 | 29,388 | 57,759 | 2,703,880 | 2.14 |
| Greece(a,c) | 980 | 895 | 1,874 | 958,678 | 69 | 939 | 589 | 1,529 | 142,736 | 1.07 |
| Hungary(a,c) | 794 | 784 | 1,578 | 1,398,428 | 71 | 1,110 | 779 | 1,889 | 114,038 | 1.66 |
| Iceland(a,c) | 1,080 | 3,218 | 4,298 | 24,217 | 75 | 26 | 58 | 85 | 17,264 | 0.49 |
| Ireland(a,c) | 835 | 3,782 | 4,617 | 143,797 | 76 | 120 | 416 | 536 | 277,689 | 0.19 |
| Israel(a,c) | 933 | 2,078 | 3,010 | 226,074 | 76 | 211 | 358 | 569 | 279,879 | 0.20 |
| Italy(a,c) | 1,084 | 1,574 | 2,657 | 5,199,501 | 68 | 5,634 | 5,578 | 11,212 | 1,398,664 | 0.80 |
| Kazakhstan |  |  |  | 2,231,579 | 86 |  |  |  | 126,332 |  |
| Kyrgyzstan |  |  |  | 729,984 | 89 |  |  |  | 6,169 |  |
| Latvia(a,c) | 822 | 839 | 1,661 | 179,842 | 70 | 148 | 106 | 254 | 23,883 | 1.06 |
| Lithuania(a,c) | 876 | 916 | 1,792 | 307,445 | 71 | 269 | 199 | 468 | 38,074 | 1.23 |
| Luxembourg(a,c) | 671 | 5,264 | 5,935 | 62,851 | 78 | 42 | 257 | 299 | 48,557 | 0.62 |
| Montenegro |  |  |  | 70,525 | 76 |  |  |  | 3,854 |  |
| Netherlands(a,c) | 1,266 | 2,453 | 3,719 | 3,202,074 | 70 | 4,055 | 5,531 | 9,586 | 632,951 | 1.51 |
| North Macedonia |  |  |  | 416,255 | 79 |  |  |  | 8,766 |  |
| Norway(a,c) | 1,315 | 3,539 | 4,854 | 442,525 | 73 | 582 | 1,137 | 1,719 | 281,597 | 0.61 |
| Poland(a,c) | 806 | 734 | 1,540 | 4,643,452 | 74 | 3,742 | 2,516 | 6,258 | 414,498 | 1.51 |
| Portugal(a,c) | 1,191 | 1,090 | 2,282 | 942,919 | 69 | 1,123 | 708 | 1,831 | 166,887 | 1.10 |
| Republic of Moldova |  |  |  | 640,739 | 78 |  |  |  | 8,325 |  |
| Romania(a,c) | 718 | 606 | 1,324 | 3,681,339 | 72 | 2,643 | 1,604 | 4,247 | 174,559 | 2.43 |
| Russian Federation |  |  |  | 24,588,952 | 77 |  |  |  | 1,177,395 |  |
| Serbia |  |  |  | 1,715,019 | 73 |  |  |  | 35,823 |  |
| Slovakia(a,c) | 870 | 906 | 1,776 | 986,737 | 76 | 858 | 677 | 1,535 | 73,518 | 2.09 |
| Slovenia(a,c) | 1,065 | 1,216 | 2,281 | 453,705 | 71 | 483 | 394 | 877 | 37,782 | 2.32 |
| Spain(a,c) | 1,142 | 1,383 | 2,524 | 5,243,633 | 72 | 5,986 | 5,233 | 11,219 | 969,613 | 1.16 |
| Sweden(a,c) | 1,358 | 2,427 | 3,786 | 834,003 | 68 | 1,133 | 1,382 | 2,515 | 371,261 | 0.68 |
| Switzerland(a,c) | 1,411 | 3,932 | 5,343 | 2,179,486 | 72 | 3,076 | 6,209 | 9,285 | 501,642 | 1.85 |
| Türkiye |  |  |  | 3,559,063 | 83 |  |  |  | 528,462 |  |
| Ukraine |  |  |  | 8,825,173 | 76 |  |  |  | 107,011 |  |
| United Kingdom(c) | 1,562 | 1,998 | 3,559 | 2,058,573 | 71 | 3,215 | 2,939 | 6,154 | 1,986,804 | 0.31 |
| Uzbekistan |  |  |  | 4,512,226 | 89 |  |  |  | 41,660 |  |
| **Total** |  |  |  |  |  | **88,512** | **95,477** | **183,989** | **13,921,550** | **1.32** |

D) Restless Legs Syndrome

| EAN member country | Annual direct (a) costs per patient (€ PPP 2019) | Annual indirect (c) costs per patient (€ PPP 2019) | Annual total costs per patient  (€ PPP 2019) | Number of prevalent cases 2019 (adult population, 20+ years) | % working age (20-64 years) | Annual direct (a) costs per country (million € PPP 2019) | Annual indirect (c) costs per country (million € PPP 2019) | Annual total costs per country (million € PPP 2019) | GDP 2019 (million € PPP 2019) | Annual total costs related to GDP (% of GDP 2019) |
| --- | --- | --- | --- | --- | --- | --- | --- | --- | --- | --- |
| Albania |  |  |  | 61,650 | 81 |  |  |  | 10,710 |  |
| Armenia |  |  |  | 67,718 | 83 |  |  |  | 9,471 |  |
| Austria(a,c) | 2,897 | 6,518 | 9,414 | 214,859 | 77 | 622 | 1,073 | 1,695 | 309,190 | 0.55 |
| Azerbaijan |  |  |  | 219,448 | 91 |  |  |  | 33,500 |  |
| Belarus |  |  |  | 222,377 | 80 |  |  |  | 44,791 |  |
| Belgium(a,c) | 2,958 | 6,071 | 9,029 | 265,962 | 75 | 787 | 1,218 | 2,005 | 372,618 | 0.54 |
| Bosnia and Herzegovina |  |  |  | 81,024 | 78 |  |  |  | 14,049 |  |
| Bulgaria |  |  |  | 168,920 | 74 |  |  |  | 47,924 |  |
| Croatia(a,c) | 1,937 | 1,996 | 3,933 | 102,851 | 75 | 199 | 153 | 352 | 43,343 | 0.81 |
| Cyprus(a,c) | 1,946 | 3,829 | 5,775 | 31,227 | 82 | 61 | 98 | 159 | 18,042 | 0.88 |
| Czechia(a,c) | 2,175 | 3,080 | 5,256 | 253,908 | 75 | 552 | 584 | 1,136 | 175,623 | 0.65 |
| Denmark(a,c) | 2,764 | 7,757 | 10,521 | 134,989 | 75 | 373 | 782 | 1,155 | 240,956 | 0.48 |
| Estonia(a,c) | 1,868 | 3,049 | 4,918 | 31,103 | 75 | 58 | 71 | 129 | 21,614 | 0.60 |
| Finland(a,c) | 2,541 | 6,330 | 8,871 | 130,934 | 72 | 333 | 593 | 926 | 186,726 | 0.50 |
| France(a,c) | 3,070 | 5,271 | 8,341 | 1,506,723 | 74 | 4,625 | 5,844 | 10,469 | 1,897,662 | 0.55 |
| Georgia(a,c) |  |  |  | 82,222 | 79 |  |  |  | 12,149 |  |
| Germany | 3,468 | 5,822 | 9,290 | 2,073,784 | 74 | 7,192 | 8,906 | 16,098 | 2,703,880 | 0.60 |
| Greece(a,c) | 2,176 | 2,492 | 4,668 | 250,969 | 73 | 546 | 454 | 1,000 | 142,736 | 0.70 |
| Hungary(a,c) | 1,763 | 2,185 | 3,948 | 233,985 | 75 | 412 | 384 | 796 | 114,038 | 0.70 |
| Iceland(a,c) | 2,398 | 8,963 | 11,361 | 7,670 | 80 | 18 | 55 | 73 | 17,264 | 0.43 |
| Ireland(a,c) | 1,854 | 10,534 | 12,389 | 107,085 | 80 | 199 | 905 | 1,104 | 277,689 | 0.40 |
| Israel(a,c) | 2,071 | 5,786 | 7,858 | 179,928 | 81 | 373 | 847 | 1,219 | 279,879 | 0.44 |
| Italy(a,c) | 2,407 | 4,383 | 6,790 | 1,484,602 | 72 | 3,573 | 4,694 | 8,267 | 1,398,664 | 0.59 |
| Kazakhstan |  |  |  | 362,751 | 89 |  |  |  | 126,332 |  |
| Kyrgyzstan |  |  |  | 117,851 | 92 |  |  |  | 6,169 |  |
| Latvia(a,c) | 1,826 | 2,336 | 4,162 | 45,644 | 74 | 83 | 79 | 163 | 23,883 | 0.68 |
| Lithuania(a,c) | 1,945 | 2,551 | 4,496 | 67,210 | 75 | 131 | 129 | 260 | 38,074 | 0.68 |
| Luxembourg(a,c) | 1,491 | 14,660 | 16,151 | 14,580 | 82 | 22 | 174 | 196 | 48,557 | 0.40 |
| Montenegro |  |  |  | 14,082 | 81 |  |  |  | 3,854 |  |
| Netherlands(a,c) | 2,812 | 6,831 | 9,643 | 401,649 | 75 | 1,130 | 2,063 | 3,192 | 632,951 | 0.50 |
| North Macedonia |  |  |  | 51,770 | 83 |  |  |  | 8,766 |  |
| Norway(a,c) | 2,921 | 9,857 | 12,777 | 122,823 | 77 | 359 | 937 | 1,296 | 281,597 | 0.46 |
| Poland(a,c) | 1,790 | 2,044 | 3,833 | 921,483 | 78 | 1,649 | 1,465 | 3,114 | 414,498 | 0.75 |
| Portugal(a,c) | 2,646 | 3,037 | 5,683 | 260,538 | 73 | 689 | 577 | 1,267 | 166,887 | 0.76 |
| Republic of Moldova |  |  |  | 88,040 | 82 |  |  |  | 8,325 |  |
| Romania(a,c) | 1,595 | 1,687 | 3,282 | 454,701 | 76 | 725 | 580 | 1,305 | 174,559 | 0.75 |
| Russian Federation |  |  |  | 3,368,116 | 81 |  |  |  | 1,177,395 |  |
| Serbia |  |  |  | 212,527 | 77 |  |  |  | 35,823 |  |
| Slovakia(a,c) | 1,932 | 2,523 | 4,455 | 129,699 | 79 | 251 | 260 | 511 | 73,518 | 0.70 |
| Slovenia(a,c) | 2,366 | 3,387 | 5,753 | 50,116 | 75 | 119 | 127 | 246 | 37,782 | 0.65 |
| Spain(a,c) | 2,535 | 3,851 | 6,386 | 1,109,629 | 76 | 2,813 | 3,233 | 6,046 | 969,613 | 0.62 |
| Sweden(a,c) | 3,017 | 6,761 | 9,778 | 235,735 | 73 | 711 | 1,171 | 1,883 | 371,261 | 0.51 |
| Switzerland(a,c) | 3,135 | 10,950 | 14,085 | 211,183 | 77 | 662 | 1,774 | 2,436 | 501,642 | 0.49 |
| Türkiye |  |  |  | 1,724,059 | 87 |  |  |  | 528,462 |  |
| Ukraine |  |  |  | 1,056,641 | 79 |  |  |  | 107,011 |  |
| United Kingdom(a,c) | 2,819 | 5,564 | 8,383 | 1,549,271 | 76 | 4,367 | 6,578 | 10,946 | 1,986,804 | 0.55 |
| Uzbekistan |  |  |  | 629,729 | 92 |  |  |  | 41,660 |  |
| **Total** |  |  |  |  |  | **33,634** | **45,810** | **79,444** | **13,921,550** | **0.57** |

E) Narcolepsy (Type 1 and 2)

| EAN member country | Annual direct (a) costs per patient (€ PPP 2019) | Annual indirect (c) costs per patient (€ PPP 2019) | Annual total costs per patient  (€ PPP 2019) | Number of prevalent cases 2019 (adult population, 20+ years) | % working age (20-64 years) | Annual direct (a) costs per country (million € PPP 2019) | Annual indirect (c) costs per country (million € PPP 2019) | Annual total costs per country (million € PPP 2019) | GDP 2019 (million € PPP 2019) | Annual total costs related to GDP (% of GDP 2019) |
| --- | --- | --- | --- | --- | --- | --- | --- | --- | --- | --- |
| Albania |  |  |  | 528 | 81 |  |  |  | 10,710 |  |
| Armenia |  |  |  | 580 | 83 |  |  |  | 9,471 |  |
| Austria(a,c) | 3,157 | 8,385 | 11,542 | 1,839 | 77 | 6 | 12 | 18 | 309,190 | 0.01 |
| Azerbaijan |  |  |  | 1,878 | 91 |  |  |  | 33,500 |  |
| Belarus |  |  |  | 1,903 | 80 |  |  |  | 44,791 |  |
| Belgium(a,c) | 3,224 | 7,810 | 11,034 | 2,276 | 75 | 7 | 13 | 21 | 372,618 | 0.01 |
| Bosnia and Herzegovina |  |  |  | 693 | 78 |  |  |  | 14,049 |  |
| Bulgaria |  |  |  | 1,446 | 74 |  |  |  | 47,924 |  |
| Croatia(a,c) | 2,112 | 2,567 | 4,679 | 880 | 75 | 2 | 2 | 4 | 43,343 | 0.01 |
| Cyprus(a,c) | 2,121 | 4,926 | 7,047 | 267 | 82 | 1 | 1 | 2 | 18,042 | 0.01 |
| Czechia(a,c) | 2,371 | 3,963 | 6,334 | 2,173 | 75 | 5 | 6 | 12 | 175,623 | 0.01 |
| Denmark | 3,780 | 7,489 | 11,269 | 1,155 | 75 | 4 | 6 | 11 | 240,956 | 0.00 |
| Estonia(a,c) | 2,037 | 3,923 | 5,959 | 266 | 75 | 1 | 1 | 1 | 21,614 | 0.01 |
| Finland(a,c) | 2,770 | 8,143 | 10,913 | 1,121 | 72 | 3 | 7 | 10 | 186,726 | 0.01 |
| France(a,c) | 3,346 | 6,781 | 10,127 | 12,895 | 74 | 43 | 64 | 107 | 1,897,662 | 0.01 |
| Georgia |  |  |  | 704 | 79 |  |  |  | 12,149 |  |
| Germany | 1,839 | 13,697 | 15,536 | 17,747 | 74 | 33 | 179 | 212 | 2,703,880 | 0.01 |
| Greece(a,c) | 2,372 | 3,206 | 5,578 | 2,148 | 73 | 5 | 5 | 10 | 142,736 | 0.01 |
| Hungary(a,c) | 1,921 | 2,810 | 4,732 | 2,002 | 75 | 4 | 4 | 8 | 114,038 | 0.01 |
| Iceland(a,c) | 2,614 | 11,530 | 14,144 | 66 | 79 | 0 | 1 | 1 | 17,264 | 0.00 |
| Ireland(a,c) | 2,021 | 13,552 | 15,573 | 916 | 80 | 2 | 10 | 12 | 277,689 | 0.00 |
| Israel(a,c) | 2,258 | 7,444 | 9,702 | 1,540 | 81 | 3 | 9 | 13 | 279,879 | 0.01 |
| Italy | 9,547 | 1,087 | 10,633 | 12,705 | 72 | 121 | 10 | 131 | 1,398,664 | 0.01 |
| Kazakhstan |  |  |  | 3,104 | 89 |  |  |  | 126,332 |  |
| Kyrgyzstan |  |  |  | 1,009 | 92 |  |  |  | 6,169 |  |
| Latvia(a,c) | 1,991 | 3,005 | 4,996 | 391 | 74 | 1 | 1 | 2 | 23,883 | 0.01 |
| Lithuania(a,c) | 2,120 | 3,281 | 5,401 | 575 | 75 | 1 | 1 | 3 | 38,074 | 0.01 |
| Luxembourg(a,c) | 1,625 | 18,859 | 20,485 | 125 | 82 | 0 | 2 | 2 | 48,557 | 0.00 |
| Montenegro |  |  |  | 121 | 80 |  |  |  | 3,854 |  |
| Netherlands(a,c) | 3,065 | 8,788 | 11,853 | 3,437 | 75 | 11 | 23 | 33 | 632,951 | 0.01 |
| North Macedonia |  |  |  | 443 | 83 |  |  |  | 8,766 |  |
| Norway(a,c) | 3,184 | 12,680 | 15,864 | 1,051 | 77 | 3 | 10 | 14 | 281,597 | 0.01 |
| Poland(a,c) | 1,951 | 2,629 | 4,580 | 7,886 | 78 | 15 | 16 | 32 | 414,498 | 0.01 |
| Portugal(a,c) | 2,884 | 3,907 | 6,791 | 2,230 | 73 | 6 | 6 | 13 | 166,887 | 0.01 |
| Republic of Moldova |  |  |  | 753 | 82 |  |  |  | 8,325 |  |
| Romania(a,c) | 1,738 | 2,170 | 3,908 | 3,891 | 76 | 7 | 6 | 13 | 174,559 | 0.01 |
| Russian Federation |  |  |  | 28,824 | 81 |  |  |  | 1,177,395 |  |
| Serbia |  |  |  | 1,819 | 77 |  |  |  | 35,823 |  |
| Slovakia(a,c) | 2,106 | 3,246 | 5,352 | 45 | 91 | 0 | 0 | 0 | 73,518 | 0.00 |
| Slovenia(a,c) | 2,579 | 4,357 | 6,936 | 429 | 75 | 1 | 1 | 3 | 37,782 | 0.01 |
| Spain(a,c) | 2,763 | 4,954 | 7,717 | 9,496 | 76 | 26 | 36 | 62 | 969,613 | 0.01 |
| Sweden(a,c) | 3,288 | 8,698 | 11,986 | 2,017 | 74 | 7 | 13 | 20 | 371,261 | 0.01 |
| Switzerland(a,c) | 3,417 | 14,087 | 17,504 | 1,807 | 77 | 6 | 20 | 26 | 501,642 | 0.01 |
| Türkiye |  |  |  | 14,754 | 87 |  |  |  | 528,462 |  |
| Ukraine |  |  |  | 9,043 | 79 |  |  |  | 107,011 |  |
| United Kingdom(a,c) | 3,073 | 7,158 | 10,231 | 13,259 | 76 | 41 | 72 | 113 | 1,986,804 | 0.01 |
| Uzbekistan |  |  |  | 5,389 | 92 |  |  |  | 41,660 |  |
| **Total** |  |  |  |  |  | **366** | **539** | **905** | **13,921,550** | **0.01** |

Tables reporting 2019 country-level prevalence and per-patient and national costs for insomnia (6A), REM sleep behaviour disorder (6B), obstructive sleep apnoea (6C), restless legs syndrome (6D), and narcolepsy (6E). Also documented here are the total national costs as a percentage of Gross Domestic Product (GDP). Letters in parentheses after country names denote imputed values (a: direct costs; c: indirect costs). Costs are expressed as 2019 purchasing power parity (PPP) EUR.
